# Revisiting the Feasibility of Public Key Cryptography in Light of IIoT Communications

**DOI:** 10.3390/s22072561

**Published:** 2022-03-27

**Authors:** Jasone Astorga, Marc Barcelo, Aitor Urbieta, Eduardo Jacob

**Affiliations:** 1 Department of Communications Engineering, Faculty of Engineering, University of the Basque Country UPV/EHU, Plaza Ingeniero Torres Quevedo 1, 48013 Bilbao, Spain; eduardo.jacob@ehu.eus; 2 Ikerlan Technology Research Centre, Basque Research and Technology Alliance (BRTA), P° J.M. Arizmendiarrieta 2, 20500 Arrasate-Mondragon, Spain; mbarcelo@ikerlan.es (M.B.); aurbieta@ikerlan.es (A.U.)

**Keywords:** ABE, blockchain, DTLS, IIoT, PKI, X.509

## Abstract

Digital certificates are regarded as the most secure and scalable way of implementing authentication services in the Internet today. They are used by most popular security protocols, including Transport Layer Security (TLS) and Datagram Transport Layer Security (DTLS). The lifecycle management of digital certificates relies on centralized Certification Authority (CA)-based Public Key Infrastructures (PKIs). However, the implementation of PKIs and certificate lifecycle management procedures in Industrial Internet of Things (IIoT) environments presents some challenges, mainly due to the high resource consumption that they imply and the lack of trust in the centralized CAs. This paper identifies and describes the main challenges to implement certificate-based public key cryptography in IIoT environments and it surveys the alternative approaches proposed so far in the literature to address these challenges. Most proposals rely on the introduction of a Trusted Third Party to aid the IIoT devices in tasks that exceed their capacity. The proposed alternatives are complementary and their application depends on the specific challenge to solve, the application scenario, and the capacities of the involved IIoT devices. This paper revisits all these alternatives in light of industrial communication models, identifying their strengths and weaknesses, and providing an in-depth comparative analysis.

## 1. Introduction

Industry 4.0 is intended to become the fourth industrial revolution. Fostered by digitalization and information and communication technologies, manufacturing systems, supply chain management and decision-making procedures will become smarter and more autonomous. This will result in disruptive industrial processes and factories. In order to achieve this goal, large-scale data gathering becomes an essential pillar, involving the deployment of Industrial IoT (IIoT) devices [1].

The realization of Industry 4.0 scenarios entails that industrial data and processes are no longer bounded to the limits of the factory. In such scenarios, the devices and applications deployed in the industrial plant need to communicate with external entities, such as X-as-a-Software services implemented in cloud providers or applications located in the premises of suppliers, service providers, etc. Such an approach opens the door to an immeasurable number of new opportunities, but it also results in the exposure of industrial systems to new potential security threats and attacks originated in the Internet. While the security issues related to the connection of traditional devices (such as PCs, laptops, servers or smart phones) to the Internet have been studied for years, industrial applications and devices are new to this world. Additionally, traditional security mechanisms are not always directly exportable to the industrial environments due to particular characteristics of these environments such as the use of specific, and sometimes proprietary, industrial communication protocols and the long lifetime of industrial systems, which might implement obsolete software and operating systems, etc.

In this context, the case of IIoT devices is especially critical, because these devices frequently support crucial processes. Therefore, the modification of the data they provide or the unavailability of these data may have an important negative impact on the supported manufacturing processes, such as the faulty manufacturing of products or stopping a manufacturing line. All of these issues directly imply important economical losses. However, the protection of IIoT devices is a challenging issue. Apart from the previously mentioned difficulties common to all industrial systems, they also imply additional challenges associated with their small memories and processing capabilities and the fact that they frequently operate on batteries, make use of wireless communication mechanisms, and are deployed in places where human access is difficult (for example, due to exposure to extreme conditions in manufacturing processes).

In the Internet, digital certificates and public key cryptography have emerged as the backbone of scalable and reliable security mechanisms. However, the implementation of these mechanisms in IIoT environments entails important challenges. Public key cryptography is by nature highly resource consuming and, therefore, it does not fit the tiny memories and processors available in small IIoT devices. In this regard, security mechanisms based on symmetric-key cryptography are better suited to the characteristics of IIoT devices, but they lack scalability and, therefore, they are not useful to support the open, heterogeneous and flexible communication patterns required by Industry 4.0 processes.

**Figure 1:**
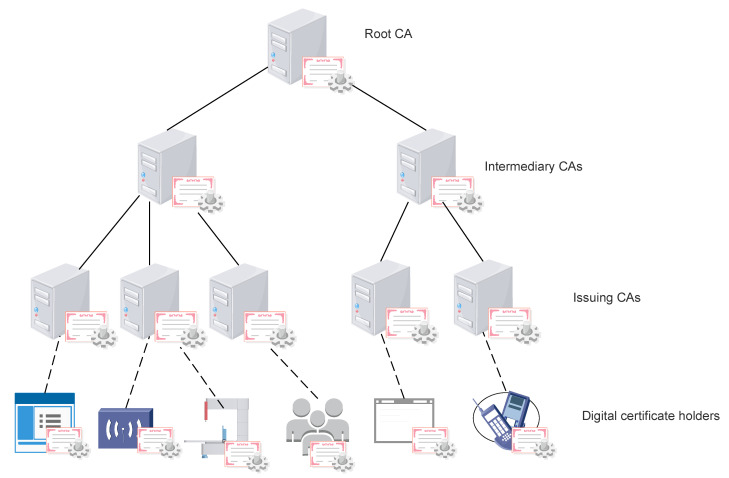
Traditional Certification Authority hierarchy.

Apart from the issues associated with the hardware limitations of the IIoT devices to be protected, the use of digital certificates and public key cryptography also entails problems inherent to the centralized nature of the currently used Public Key Infrastructures (PKI). On the one hand, traditional Certification Authority (CA)-based centralized PKIs are subject to lack of trust suspicions. As it will be explained in depth in Section 5, in the current hierarchical CA architecture, based on a tree structure as depicted in Figure 1, the security of the whole system depends on the security of the high-level CAs. Therefore, if any element of the tree is compromised, the rest of the branches hanging from the compromised element are automatically compromised too. Security vulnerabilities can be of a technical nature, such as in the case of malicious attacks or sloppy configurations, but they can also be the result of organizations controlling CAs wanting to snoop into citizens’ communications. On the other hand, the expected massive deployment of IIoT devices raises new challenges for the current centralized CA architectures which will struggle to support all these new devices.

In this context, the objective of this survey paper is to study the challenges that the implementation of public key cryptographic mechanisms involves for IIoT devices and to analyze alternative approaches to address them. The novelty of this survey paper lies in both the type of considered mechanisms and the application scenario. There are previous papers that survey the implementation of several types of cryptosystems [2,3] and authentication mechanisms [4,5] in IoT scenarios. However, neither of them studies the different issues associated with the implementation of public key mechanisms, and besides this, no previous work considers the specific context of industrial scenarios. In this regard, there are some issues that are common to all IoT devices, and others specific to the industrial environments and the used protocols and communication architectures. For this reason, after introducing the characteristics of industrial communications, the paper is structured so that in Section 3, Section 4 and Section 5, the limitations common to all IoT devices are first studied and alternative approaches assessed. Then, this analysis is revisited in light of the industrial communication protocols and environments.

The rest of the paper is structured as follows: Section 2 presents the most common communication protocols in industrial scenarios and the implemented security mechanisms and extracts common characteristics. Next, Section 3 studies the mechanisms proposed so far to make Datagram Transport Layer Security (DTLS) connections feasible in IoT devices and analyzes the applicability of the studied alternatives in industrial environments. Section 4, in turn, assesses the possibility of using Attribute-Based Encryption (ABE) as an alternative to traditional public key encryption in environments that involve IoT devices, and more specifically, in industrial communication scenarios. Similarly, Section 5, studies the applicability of blockchain to replace the current centralized PKIs and its feasibility in industrial contexts. Then, Section 6 presents an in-depth comparative analysis of all the studied approaches and finally, Section 7 gathers the main conclusions of the paper.

## 2. Industrial Communication Protocols

In this section, the specificities of industrial communication protocols are presented in order to define the characteristics of industrial communication scenarios.

**Figure 2:**
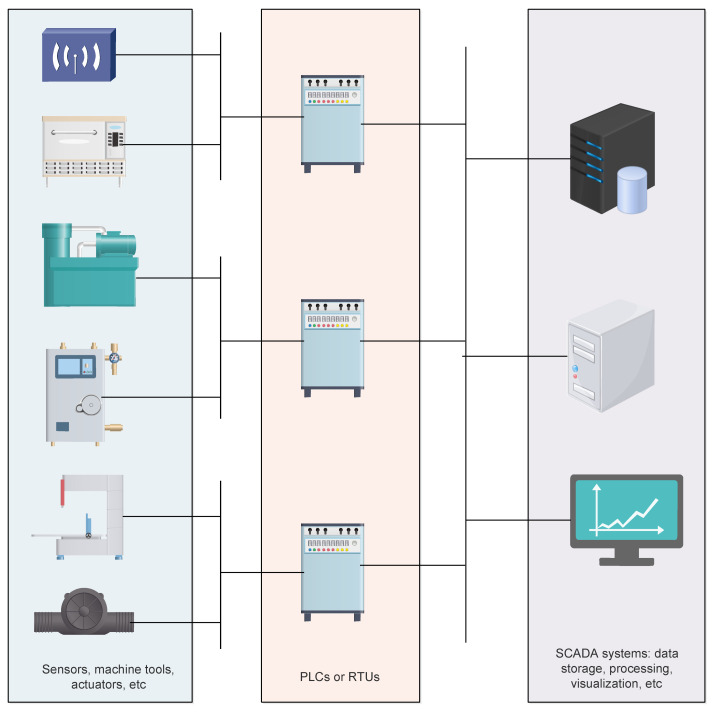
Basic architecture of a SCADA system.

Overall, industrial communication networks are mainly based on Supervisory Control and Data Acquisition (SCADA) systems aimed at controlling industrial processes by monitoring, gathering and processing real-time data. SCADA systems consist of software and hardware elements where usually Programmable Logic Controllers (PLCs) or Remote Terminal Units (RTUs) communicate with a set of elements deployed in the industrial plant, such as machine tools, sensors, actuators, etc. The PLCs and RTUs implement SCADA software to route the information gathered from these elements to computers running SCADA software, which processes and displays the data in a way that is easily understandable by human operators and helps them to make important decisions. Figure 2 shows the overall architecture of a SCADA system.

Among the most popular protocols currently used in TCP/IP-based SCADA systems, the following can be highlighted: AMQP, MQTT, XMPP, Modbus TCP, OPC UA and CoAP. Next, these protocols are briefly described.

The first versions of the Advanced Message Queuing Protocol (AMQP) (referred to as AMQP 0-9-1) constitute a messaging protocol strongly based on the use of middleware brokers, where these brokers route messages received from publishers to consumers. However, the latest version of AMQP, known as AMQP 1.0 [6], comprises important differences with respect to previous specifications of the protocol, being the most relevant one due to the fact that it does not define a broker. This latest version of the protocol is the only one standardized by OASIS and ISO/IEC, and it focuses on the messaging layer, that is, on how data is transferred on the wire. In this way, the protocol becomes open to alternative implementations regarding message routing and handling, and thus, interoperability and security are easier to achieve. Regarding security, AMQP supports encryption only or encryption and authentication, based on X.509 [7] certificates, by means of Transport Layer Security (TLS) [8]. It also supports multiple authentication mechanisms by means of Simple Authentication and Security Layer (SASL) [9], such as anonymous, plaint-text and MD5-diggest. The full list of available SASL mechanisms is available at [10].

Message Queuing Telemetry Transport (MQTT) is a lightweight and easy-to-implement messaging protocol, open OASIS [11] and ISO standard (ISO/IEC 20922) [12], that works according to a publish-subscribe paradigm. The MQTT protocol defines two types of entities: clients and broker. Usually, an MQTT broker is a server and MQTT clients are the devices that connect to this server either to send data (publish) or to receive it (subscribe). To enable the operation of the MQTT protocol, sessions are established between the broker and the clients. Although MQTT implements an authentication phase during the session establishment, the security level achieved with this functionality is very limited since usernames and passwords are sent in cleartext. Therefore, MQTT is usually implemented over SSL/TLS sessions. Nevertheless, MQTT does not implement any mechanism to control who can publish information in the broker or to allow a message receiver to authenticate the origin of a message, unless that information is contained in the actual message payload. Currently, these security features, are implemented when needed on top of MQTT by means of proprietary, out-of-band messages, which results in increasing the code footprint and making implementations more complex.

Extensible Messaging Presence Protocol (XMPP) is a standard protocol of the IETF [13] designed for the streaming of XML elements over a network, in order to achieve interactive exchange of messages and presence information (such as “available”, “offline”, “busy” and any other defined by the user with the presence stanza) in a close to real-time fashion. This protocol is based on a decentralized client-server architecture and it works according to a publish-subscribe mechanism. Clients have unique names and communicate with other clients by means of an intermediary XMPP server. In order to provide this routing functionality between source and destination clients belonging to different domains, XMPP servers can also communicate among them. Apart from XMPP clients and servers, XMPP gateways may also exist, with the aim of translating between XMPP and other messaging protocols (for example, SMS or SMTP). For the creation of XMPP streams between a given client and server, XMPP sessions must be established, which include an authentication phase. XMPP supports different types of authentication mechanisms, including plain-text authentication, MD5 message-diggest based authentication, Kerberos or the use of special tokens. After this authentication phase, TLS is used to encrypt XMPP streams.

Modbus [14] was originally designed by Modicon in 1979 to control and gather data from its range of PLCs. Being a public and easy to use protocol that requires little development, Modbus was rapidly widespread, and it became a de facto standard for industrial communications. Currently, it is the most widely used protocol for the interconnection of industrial electronic devices and in order to allow running Modbus over TCP/IP networks, Modbus TCP was created. Modbus operates in a request–response mode based on a master-slave architecture, where the master is always responsible for initiating the communication, sending a request, and waiting for the response from the slave device. Modbus has a secure version known as Modbus TCP Security. In this secure version, the protocol header is encapsulated over TLS, providing the capacity to implement peer authentication and authorization, as well as frame confidentiality and integrity protection. Authentication and authorization are achieved by the use of X.509v3 certificates with extensions to convey roles, which are later used to implement Role Based Access Control (RBAC). The authorization rules are specifically designed by each user.

OLE for Process Control Unified Architecture (OPC UA) is an improvement of the OPC protocol, which was designed to communicate data securely in industry as well as in other sectors. It is an open standard [15] platform developed by the OPC Foundation. OPC UA can work on a client/server architecture or on a publisher/subscriber model and it has been designed so that it can be transported over multiple existing protocols, such as SOAP, HTTP, HTTPS or directly TCP. The OPC UA data model is based on “objects”, where an object can be anything from a simple piece of information to a whole procedure, a complex system or an entire plant. OPC UA has been designed with security in mind and has defence in-depth implemented in the different layers of the architecture. Security in OPC UA can be divided into three major blocks: Transport Layer Security, Communication Layer Security and Application Layer Security. At the transport layer, TCP is used and security is based on standard TLS. On top of this, the communication layer implements application authentication and message integrity and confidentiality. Application authentication is based on PKI infrastructures, where clients and servers exchange X.509 certificates. Once clients and servers establish a trusted relationship, they build a secure channel at the communication layer. On top of this secure channel, OPC UA implements a user authentication mechanism based on a user token. The token format is different depending on the implemented application level authentication mechanism. Currently OPC UA supports four types of user authentication: anonymous, username/pass, x.509 v3 certificate, binary token issued by an external authentication service. This information is then used to enforce access control mechanisms. Messages can also be encrypted or integrity protected at the application layer. This mechanism is based on group keys shared by subscribers, which have to access a given secured content. Keys can be pre-shared offline or managed by a central server, such as Kerberos.

Constrained Application Protocol (CoAP) is an IoT protocol, standardized by the IETF [16], and mainly designed for Machine-To-Machine (M2M) communication. The protocol is based on an asynchronous message exchange, and it supports URIs, proxies and caching capabilities, following an approach similar to HTTP. CoAP is based on a client-server model where clients send asynchronous messages to servers and wait for a response. For these interactions GET, PUT, POST and DELETE methods are supported. CoAP runs over UDP, and therefore, DTLS is frequently used to provide security to CoAP communications. Usually, DTLS capable CoAP entities support RSA or ECDSA for authentication and AES for encryption.

Table 1 summarizes the main characteristics of the reviewed industrial communication protocols. As shown in Table 1, industrial communications include two types of very distinct communication patterns: (1) Peer to peer (client-server) architectures, where information producers and consumers establish a direct end-to-end communication. In such cases, any operation, such as providing certain information or performing an action, is only started as a response to a query sent by the client peer. (2) Brokered communication architectures, where information producers and consumers do not establish a direct end-to-end communication. Instead, a broker is used as an intermediary. Therefore, publishers feed information to the broker and consumers read this information from the broker. In this case, two end-to-end communications are established, one between the publisher and the broker, and another one between the broker and the consumer. In the case of brokered communications, two operational modes are possible: *push* operational mode, where messages are pushed to all subscribed consumers, and *pull* operational mode, where the broker only sends messages to consumers upon request from their part.

Regarding security, most industrial communication protocols rely on transport layer security (TLS or DTLS depending on the transport layer protocol being used) based on public key cryptography and digital certificates. In the case of brokered communications, as two independent transport layer communications are implemented to allow routing of messages from publisher to subscriber by means of the broker, protecting information at the transport layer means that this information will be unprotected for some time at the intermediary broker.

Taking into account that most industrial communication protocols rely on TLS/DTLS for security, specifically DTLS in the case of wireless UDP-based IoT devices, the next section will analyze how this protocol can be efficiently implemented in IoT environments. Then, the specific concerns regarding IIoT scenarios will be considered. Additionally, as TLS/DTLS handshake is most frequently based on the exchange of X.509 digital certificates, the impact of these certificates and the PKIs used to support their life cycle management in IoT and IIoT scenarios will also be studied.

## 3. Affordable DTLS for IIoT Devices

As already mentioned, current solutions to enable scalable authentication services worldwide rely heavily on CA-based centralized PKIs. This is also the basis of the well-known TLS protocol [8]. DTLS has been created as an attempt to adapt to the IoT world and the widespread TLS protocol, and thus, to provide a standard security layer to IoT application level protocols. In fact, DTLS has already become a building block of IoT security. Nevertheless, it must be considered that the security level provided by both TLS and DTLS depends on the security level of the implemented cryptosystems. For this reason, currently robust cryptosystems tailored to the efficiency needs of IoT devices are being proposed, such as [17,18]. Additionally, quantum computing poses a special threat to TLS and DTLS communications, especially Shor’s [19] and Grover’s [20] algorithms. In order to face the threat of quantum computing to current security mechanisms, NIST (National Institute of Standards and Technology) proposed a contest to standardize post-quantum cryptographic algorithms. At this time, the third round is in progress with only 15 candidates from the 69 starting ones, looking forward to the fourth and last round of the contest.

In the rest of this section, first, the operation of the latest version of DTLS (DTLS 1.3) is explained and then, the most relevant approaches to make DTLS affordable for resource-deprived IoT devices are analyzed.

### 3.1. DTLS Fundamentals

DTLS aims to be a protocol equivalent to TLS, but over datagram-based communications, such as UDP or Datagram Congestion Control Protocol (DCCP). Therefore, DTLS is similar to TLS, but it has to solve some problems inherent to datagram-based communications, such as packet losses and out-of-order arrival of packets. To deal with packet losses, DTLS implements a mechanism based on timers and retransmissions, which are triggered whenever the corresponding response has not been received before the timer expires. With respect to reordering, this issue is addressed by using sequence numbers and maintaining a sequence-related state in the communicating peers. When one of the peers receives a message, the recipient peer compares the sequence number within the message with the expected sequence number. If the received message is the next message expected by the peer, the message is processed. If the received message is a future message, the recipient peer stores the received message for later processing once all previous messages have been received. If the received message is an old message, the message is discarded.

**Figure 3:**
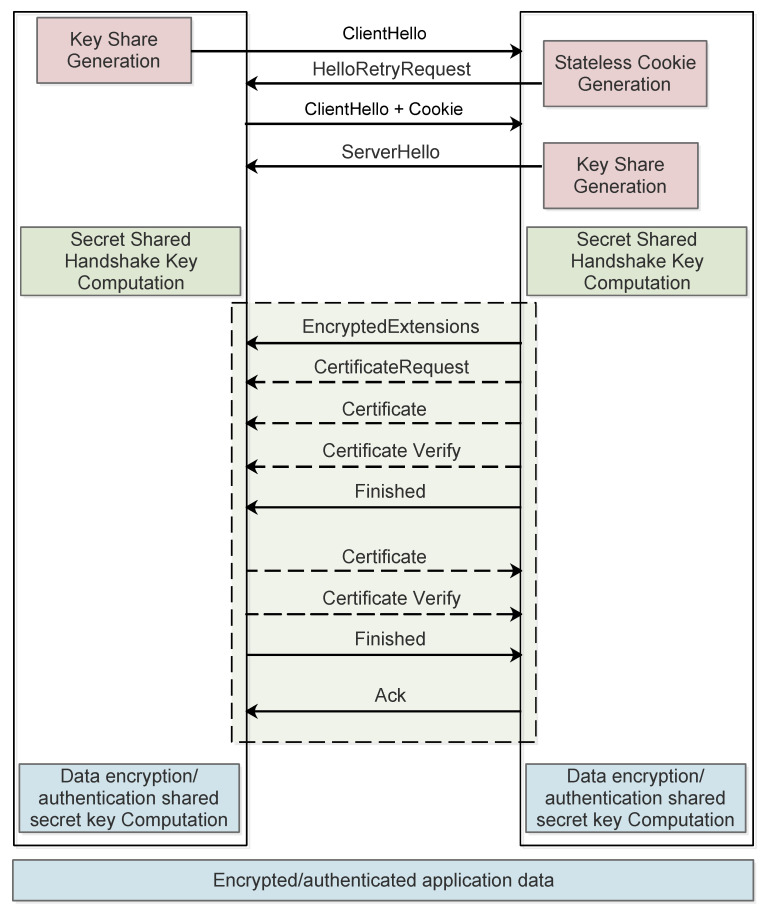
Message exchange of the DTLS 1.3 handshake protocol.

DTLS follows the same structure as TLS and it is also based on a two-layer architecture, with two main protocols: the DTLS handshake protocol and the DTLS record protocol. The handshake protocol provides mutual authentication as well as mechanisms for the negotiation of security settings and cryptographic keys. Then, the DTLS record protocol makes use of the cryptographic suites and material negotiated in the handshake phase to encrypt and authenticate, by means of Message Authentication Codes (MACs), individual packets. Figure 3 shows the DTLS 1.3 handshake exchange in which mutual authentication is achieved by means of digital certificates and a mechanism based on Diffie–Hellman is used for key exchange.

DTLS version 1.3 implies important differences with respect to previous versions of the protocol. On the one hand, it allows improving efficiency by using shorter messages; on the other hand, it also enhances security, by removing weak cryptographic primitives and including new stronger security mechanisms.

As shown in Figure 3, the DTLS 1.3 handshake is based on a sequence of message flights. To start the DTLS handshake, the client sends a *ClientHello* message to the server. In this message, the client specifies the cipher suites it supports, its public key parameters and key shares for the Diffie–Helmman key exchange. Optionally, the server may respond with a *HelloRetryRequest* message, which contains a fresh stateless cookie generated by the server in real time. When the client receives this message, it must retransmit the *ClientHello*, but this time with the cookie added as an extension. When receiving the retransmission of the *ClientHello*, the server verifies the cookie and only if the received cookie is correct, the server responds with a *ServerHello* message, which conveys the server’s key share and specifies the selected security suite. The message exchange associated with the stateless cookie avoids DoS attacks with spoofed IP addresses. However, it is not effective to protect from DoS attacks originated from valid IP addresses. At this point, key shares have been exchanged, and therefore, client and server compute a secret shared key based on the Diffie–Hellman protocol. This key is used to encrypt the remaining messages of the handshake.

The server proceeds with an *EncryptedExtensions* message, specifying additional security settings; an optional *CertificateRequest* message, if it wants to request a certificate from the client for client authentication; a *Certificate* message, with its own digital certificate; a *CertificateVerify* message, to authenticate the server’s side of the key exchange, and finally, a *Finished* message to end the flight of messages and to confirm the security of the handshake and the encrypted channel.

As a response to this flight of messages, the client sends a *Certificate* message, with its own digital certificate, for authentication purposes; a *CertificateVerify* message to verify the client’s side of the key exchange; and a *Finished* message to end the message flight, authenticate the handshake and confirm the security of the encrypted channel, respectively. As DTLS is carried over UDP, an unreliable transport protocol, the server finishes the handshake message exchange with an *Ack* message that acknowledges the correct reception of the information sent by the client. This *Ack* message ends the handshake exchange and from this point on, the client and server can use the DTLS record protocol parametrized with the negotiated security settings and cryptographic keys in order to ensure the confidentiality and authenticity of the exchanged application level data.

### 3.2. DTLS Delegation-Based Alternatives

The use of digital certificates as authentication mechanism for the establishment of DTLS channels presents important challenges for resource-deprived IoT devices. Specifically, the most costly operations are the validation of certificate chains and the execution of the OCSP protocol to check if a certificate has been revoked. Additionally, the execution of public key cryptographic operations is also a challenge for the most resource-deprived IoT devices, such as motes. The most common approach to address these challenges is to delegate the execution of these costly operations to resource-richer devices. Taking into account the broad scope of available IoT devices and use contexts, different levels of delegation have been proposed, which also have different implications from a security point of view.

The most efficient alternatives [21,22,23,24,25,26,27,28], are based on delegating the whole DTLS handshake on a resource-richer trusted entity and then, leveraging the session resumption feature provided by DTLS in order to convey the session negotiated by the resource-rich entity to the IoT device.

More specifically, in 2013 Granjal et al. [21] proposed an architecture where a 6LoWPAN [29] Border Router (6LBR) is introduced in-between a resource-deprived IoT device and an Internet entity to release the IoT device from dealing with certificates and performing public key cryptographic operations. For this aim, two DTLS handshake processes are carried out in parallel: a certificate-based DTLS handshake between the Internet entity and the 6LBR and a second DTLS handshake based on Pre-Shared Keys (PSKs), between the IoT entity and the 6LBR. However, the 6LBR remains transparent for both endpoints (Internet and IoT entities) and from the point of view of each of them, the handshake is performed end-to-end with the other endpoint.

Similarly, in [22], Hummen et al. proposed to delegate the full DTLS handshake to a trusted entity known as the delegation server. After establishing the DTLS session, the delegation server transfers the session ticket to the IoT device, which resumes the DTLS session to establish new cryptographic material. A similar approach is presented in [23], where the border gateway between the IoT network and the Internet is used to delegate the DTLS handshake.

Likewise, in [24] the authors introduce the concept of Secure Service Manager, consisting of three logical entities: a Host Server, a Resource Directory and a DTLS handshake delegator (HS Delegator). A client that wants to access data in an IoT device, queries the Host Server, which determines if DTLS handshake delegation must be performed. In an affirmative case, the session is redirected to the HS Delegator which performs the whole DTLS handshake on behalf of the IoT device. When the handshake finishes, the HS Delegator sends the session ID to the IoT device , as well as the DTLS version and cryptographic material. An extension to this work is presented in [25], where the authors propose to integrate the DTLS handshake delegation with a secure bootstrapping scheme and provide an implementation and evaluation of the proposed system.

Progressing with this work, in [26], the authors propose to delegate the DTLS handshake to the cloud. For this aim, the authors introduce a new concept of IoT devices consisting of two logical entities: the physical thing, which is the actual device; and the virtual thing, a software module deployed in a cloud environment which aids the physical thing in complex security-related computations, namely, the DTLS handshake.

Following the same approach, ref. [27] introduces an architecture based on back-end offloads (BeO nodes), which are powerful devices belonging to the IoT network, where security-related operations are delegated. The difference with respect to previous proposals is that in this case, both the DTLS handshake and the DTLS record are delegated to the BeO nodes.

In a more recent work [28], the authors propose a light DTLS handshake for IoT communications, implemented by means of Software Defined Networking (SDN). This proposal is based on delegating the Diffie–Hellman Key Exchange and certificate verification operations to a resource-rich SDN controller. Additionally, the controller replaces the DTLS server and performs a cookie exchange with the client and generates and distributes a symmetric key to the communicating endpoints.

All aforementioned solutions allow the release of the resource-deprived IoT devices from the highly costly DTLS handshake, but they entail important security and privacy concerns. In fact, for a full delegation of the DTLS handshake to a powerful Trusted Third Party (TTP), the IoT devices must also delegate their private keys and the TTP gets to know the private keys of all the IoT devices it acts on behalf of. This goes against the nature of private keys, which must only be known to their owner.

In order not to incur the security concerns associated with the delegation of the IoT devices’ private keys to a TTP, some works [30,31] opt for delegating only those operations that do not require knowledge of the private key. The work in [30], proposes to delegate the verification of certificate chains to a more powerful centralized server, which will then create and distribute certificate allowlists (traditionally known as whitelists) to the involved IoT devices. The proposed allowlists enumerate explicitly all accepted certificates, either using the subject name, the serial number or any other information that allows to unambiguously identify a certificate, and they are presented as an alternative to a globally accepted certification authority. This approach is not scalable and is only acceptable for small- or mid-size deployments.

In [31], the authors introduce a “security agent” in order to perform most costly operations of the DTLS handshake on behalf of the IoT device. Nevertheless, the “security agent” never gets to know the IoT device’s private key. Operations that require the IoT device’s private key are performed by the IoT device itself. However, the security agent gets to negotiate the DTLS session parameters, including the session key. When the handshake is finished, the security agent forwards the negotiated session to the IoT device, which performs a DTLS session resumption.

Taking into account the benefits and disadvantages of full and partial DTLS handshake delegations, some proposals [32,33,34] allow to adapt the amount and type of delegated operations, according to the characteristics of the protected IoT devices. Already in 2006, Fouladgar et al. [32] proposed a mechanism for delegating part of the TLS handshake from a resource-deprived IoT node to a resource-richer IoT network gateway. The authors proposed two variants of the delegation mechanism. In the first version, the IoT gateway is just partially trusted, and therefore, only the authentication phase is delegated, not the key exchange phase. In this way, the semi-trusted IoT gateway does not get to know the TLS session key. In the second version, a fully trusted IoT gateway is assumed, where the whole TLS handshake is delegated, also allowing a greater saving of resources.

In 2013, Hummen et al. [33] published some ideas about how to make feasible the use of digital certificates in resource-deprived devices. For very constrained devices, the authors propose to delegate the whole DTLS handshake to a resource-richer TTP. In the case of not so constrained devices, the authors propose the mechanisms of on-path pre-validation and session resumption. On-path pre-validation consists of the IoT gateway intercepting the DTLS handshake message and validating the certificate chain. Therefore, it is no longer necessary for IoT devices to perform this validation, and neither to implement costly protocols such as Network Time Protocol (NTP) and Online Certificate Status Protocol (OCSP).

Similarly, in [34], the authors introduce a level of flexibility in the delegation mechanism, allowing IoT devices to decide on the tasks to delegate depending on their capacity, their instantaneous workload or environmental conditions such as battery level. The tasks that could be independently delegated are ECDH keys generation, pre-master secret generation, ECDSA signature generation, ECDSA signature verification and certificate verification.

It must be noted that all the alternatives where the TTP negotiates DTLS session keys on behalf of the IoT devices suffer from security and privacy concerns. This affects all the full delegation solutions and those partial delegation approaches where the key exchange is among the delegated tasks. In such cases, the TTP gets to know all the cryptographic material created to protect the DTLS session. Therefore, it could snoop in information exchanges protected with that material thereafter and even perform a Man-In-The-Middle attack. Additionally, all the studied alternatives assume the existence of a PSK between each IoT device and the TTP in order to protect all the pair-wise communications between them. However, it is not explained how these security associations are established, and how they are supposed to take place offline.

An alternative approach is to delegate costly operations not to an external TTP, but to a local hardware module, such as a Trusted Platform Module (TPM). In [35], the authors propose to use a TPM in each IoT device in order to hold the private key of the IoT device and perform the corresponding private key cryptographic operations. The presented testbed-based performance evaluation shows that the proposal is affordable in terms of latency, energy consumption and memory footprint. This solution enhances the protection of the private key in IoT devices and removes the problems associated with trusting a third party for full or partial delegation. However, the use of a TPM implies an increase in the cost and complexity of the IoT devices.

Table 2 provides a summary of the analyzed alternatives to delegate partial or full DTLS handshake to a resource-richer party.

### 3.3. Compression-Based Alternatives

In order to make the use of certificates feasible for resource-deprived IoT devices, some authors have focused on alleviating the burden introduced in IoT networks by the lengthy encoding format of X.509 certificates. With this aim, proposed alternatives follow two main approaches: (1) remove redundant information or fields that can be inherently obtained and (2) replace human-readable codifications by more efficient binary codification.

**Figure 4:**
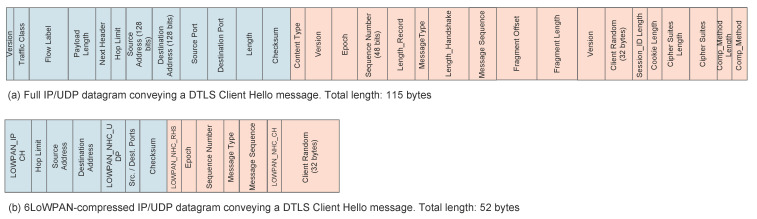
IP/UDP packet conveying a ClientHello message: (**a**) uncompressed, (**b**) compressed following the 6LoWPAN strategy.

In [36], Raza et al. propose and evaluate the performance of a mechanism to compress DTLS headers, called Lithe. The proposed solution is based on the use of 6LoWPAN and it consists of defining a new bit sequence in the 6LoWPAN Next Header Compression (NHC) header that indicates that the next header is compressed. Then, DTLS header compression also follows the 6LoWPAN approach: fields whose information can be implicitly obtained from other fields or which usually carry default values are not sent in line and default values are assumed. Similarly, the size of long fields is reduced by sending just the part that varies and assuming a default value for the part that stays immutable. As an example of the proposed approach, Figure 4 shows a DTLS *ClientHello* message compressed according to the proposed strategy. Then, in [37], Lithe is used along with a cloud platform to implement secure CoAP communications in IoT devices. Similarly, in [38], Haroon et al. combine the Lithe compression mechanism with the integration of a TTP that pre-shares keys with both the DTLS client and server. As a result, the preparation of the *ClientHello* message takes longer, but the overall DTLS handshake duration is reduced and the IoT devices are better protected against Denial of Service (DoS) attacks.

Similarly, in [39], the authors propose to use the 6LoWPAN NHC to reduce the size of DTLS headers. They specify encodings for record and handshake headers. Regarding handshake, they propose to compress *ClientHello* and *ServerHello* messages, while the rest of the DTLS handshake messages are carried uncompressed.

The work in [40] is focused on reducing the overhead introduced by currently used X.509v3 certificates, by replacing these certificates with smaller certificates, such as self-descriptive card verifiable certificates. Additionally, they also propose to replace the ASN.1 syntax used to identify subjects with IPv6 addresses, more suitable for large numbers of IoT devices. On the other hand, the authors propose also to include authorization-related attributes by means of extension fields, in order to provide authorization services.

Following the same research direction, in [41,42], Kwon et al. propose to replace traditional ASN.1 certificate codification with the Concise Binary Object Representation (CBOR) [43] format, and to apply the 6LoWPAN header compression strategy to the compression of X.509 certificate fields. More specifically, they propose to remove from X.509 certificates the fields that are common to the whole IoT network, and therefore, implicitly understandable for the IoT devices. In order to keep compatibility with the traditional X.509 certificates used in the Internet, the IoT border router is in charge of compressing traditional certificates before they enter the IoT network, and reconstructing compressed certificates in the opposite direction.

In [34], a very light new type of certificate is defined, called PKIoT. The PKIoT certificate is not standard and only works with the PKIoT architecture, where the certificate verification task is delegated to resource-richer servers. In fact, the PKIoT certificate consists just of a link to the full certificate so that the PKIoT server can obtain it for verification.

The work in [44] proposes a lightweight profile for the compression and encoding of X.509 certificates. The proposed encoding format is called XIOT and it is based on removing from X.509 certificates fields with fixed values (such as version number or signing algorithm, which is fixed by the proposal) and then encoding the resulting compressed certificate with CBOR.

In general, these solutions allow to reduce energy consumption in IoT devices because overall, they reduce the number of transmitted bytes and energy consumption due to transmission/reception operations being much greater than energy consumption due to computation. Additionally, such solutions also allow to minimize fragmentation needs in IoT networks with small MTU sizes, which improves security because protocols such as 6LoWPAN have been proved to be vulnerable to some fragmentation attacks [45]. On the other hand, the main disadvantage of these approaches is that IoT devices no longer use standard formats for digital certificates and protocol messages, and therefore, implementations are dependent on the associated translation software between standard and compressed formats. As a result, compatibility and flexibility are heavily penalized.

Table 3 summarizes aforementioned approaches for compression of DTLS protocol headers and X.509 certificates.

### 3.4. Discussion on Alternatives to Achieve Affordable DTLS for IIoT Devices

The element that currently hinders the implementation of DTLS in IoT devices is the available memory. In fact, common DTLS implementations with all the libraries necessary to run the different cryptographic suites result in big software pieces, which exceed to a large extent the memory available in most IoT devices. For this reason, the efforts to make DTLS feasible in IoT devices are directed to reduce the memory footprint of the DTLS software, as well as the dynamic memory usage during the DTLS execution, by delegating costly tasks to an external TTP. Delegation fits industrial environments involving specific and expensive equipment with long lifetimes. These equipments frequently run obsoleted software and operating systems, which do not meet the latest security specifications and which cannot be updated. The delegation of security tasks to an external and up to date entity allows the implementation of the latest security standards.

With the aim of reducing memory footprint, many proposals [21,22,23,24,25,26,27,28,30,31,32,33,34] focus on removing the most complex DTLS tasks from the DTLS code to be installed in the IoT devices. These tasks are mainly related to the DTLS handshake, which implements a much more complex state machine in comparison with the DTLS record protocol. However, the handshake is a critical part of the DTLS connection, since it involves mutual peer authentication and negotiation of cryptographic material for application data encryption and MAC computation. Therefore, the proposed approaches try to achieve a trade-off between security and performance, in terms of the amount and type of delegated DTLS tasks.

Apart from a complex and lengthy handshake process, DTLS suffers also from long protocol header structures, which are not suitable for the short frames used in IoT communications. Therefore, there are also works [34,36,39,40,41,42,44] intended to reduce the size of DTLS headers, mainly using more efficient binary codifications and following the 6LoWPAN compression approach. However, such mechanisms result in non-standard certificate and message formats. The utilization of none-standard formats is not a recommendable practice for industrial environments, where standards should always be followed in order to guarantee interoperability. Therefore, the option of compressing DTLS headers by non-standard mechanisms should be discarded in industry as much as possible.

Regarding the different communication models existing in industrial environments, in the case of brokered industrial communication architectures, no direct end-to-end connection exists between the IIoT device, acting normally as publisher, and the consumer of the industrial information (normally a Human–Machine Interface (HMI) or a SCADA system). Therefore, the most suitable solution in this case would be to opt for an approach similar to Granjal et al.’s [21] proposal, where two independent DTLS sessions with different characteristics are established. The communication between the broker and the resource expensive consumer could be protected using certificate-based DTLS, without the need of handshake delegation. On the other hand, the communication between IIoT devices and the broker should be protected using a mechanism tailored to the capacities of the protected IIoT devices, which might be PSK-based DTLS. The distribution of PSKs in this case, where all the participating entities belong to the same administrative domain, should not imply a big challenge.

In the case of client–server communication architectures, where the DTLS session is established end-to-end directly between the IIoT device and the external consumer, DTLS delegation and header compression must be enforced when the IIoT device has severe resource constraints. In this case, the best option would be to opt for a flexible delegation mechanism, such as the works proposed in [32,33,34], where the delegated operations can be adjusted depending on the specific capacities of each protected IIoT device. Regarding the entity and where to delegate handshake and compression tasks, the optimal place would be the IIoT border gateway that interconnects the IIoT network and the rest of the factory networks. This must be a trusted entity that shares a trust relationship with all IIoT devices.

In order to implement delegation and compression tasks, the IIoT gateway will usually rely on home-made and tailored software implementations, which are difficult to maintain and to keep up to date with the latest security patches. Additionally, if it is opted for a full DTLS handshake delegation, the IIoT devices’ private keys must be communicated to this IIoT border gateway. For all these reasons, special security mechanisms must be applied to protect it, such as hardening, as it becomes an especially vulnerable entity that owns factory-critical information.

## 4. Public Key Encryption in Brokered Communications

Frequently, industrial scenarios are based on the deployment of a high number of IIoT devices that pervasively gather data about the environment and about the actual manufacturing processes. These data are then consumed by multiple applications with different purposes, such as the monitoring of a specific manufacturing process or doing some business analytics. In such contexts, the protection of this information by means of traditional certificate-based public key cryptography implies performing the certificate exchange, key authentication and encryption operations individually for each endpoint. When IIoT devices are massively deployed, the protection of IIoT communications in such a traditional way could overload the IIoT network and the actual IIoT devices.

In such scenarios, alternative encryption mechanisms such as ABE, would be preferred. ABE [46] entails a novel cryptographic approach which removes the necessity of digital certificates. ABE has been created as an evolution of Identity-Based Encryption (IBE). This asymmetric cryptographic schema was proposed by Shamir in 1984 [47] and its main goal was to avoid the necessity of a centralized PKI to link identities with their corresponding public keys. The idea to achieve this goal is very simple: use as the public key the direct identity of the recipient (for example, their email address). According to this approach, anyone can send a confidential message to an intended recipient without needing to download and check digital certificates, just by using the destination’s identity as a public key. The first fully-functional IBE implementation was not available until 17 years after the idea was introduced, and it was developed by Boneh and Franklin [48]. Not long afterwards, in 2004, Amit Sahai and Brent Waters [46] developed a new cryptosystem which generalized this approach. They called their system Fuzzy Identity-Based Encryption, but it is more widely known as Attribute-Based Encryption (ABE).

In fact, ABE is a particular type of asymmetric encryption schema where public/private key pairs are not randomly generated. Instead, the public key is specifically defined according to a policy or attribute set. Then, multiple private keys are generated that would decrypt the corresponding public key, one for each of the intended recipients of the confidential communication. Therefore, ABE is an encryption mechanism that suits the characteristics of brokered communications in industrial scenarios: it removes the necessity of maintaining a centralized PKI hierarchy and it allows including an access policy in the encrypted data, so that the same encrypted data can be decrypted by multiple destinations that match the access policy.

### 4.1. Fundamentals of ABE

ABE generalizes the basic concept introduced by IBE, where the public key used to encrypt confidential data is somehow linked to the identity of the intended recipient. The fundamental concept of ABE is to use as the encryption public key a set of attributes that would define an intended group of recipients. Any user with a given number of the required attributes is able to obtain the necessary private key to decrypt the message. Therefore, ABE is especially suitable for situations where confidential information must by distributed to a group of users, since it avoids the necessity of distributing and managing group keys, and the issues linked to them, such as revocation when a user is no longer a member of the entitled group.

Currently, the concept of ABE gathers two different approaches: Key-Policy Attribute-Based Encryption (KP-ABE) [49] and Ciphertext-Policy Attribute-Based Encryption (CP-ABE) [50]. Both alternatives use an access tree to define an access policy using attributes. In KP-ABE, the users’ private keys are associated with an access policy, which may be any monotonic tree, and the public key is associated with an attribute set. A user is able to decrypt the ciphertext if its access policy is satisfied by the attributes embedded in the ciphertext. Therefore, KP-ABE has one big disadvantage: the entity encrypting a confidential message cannot decide on the access policy to this message, and therefore, on who will be able to decrypt the confidential message. As a consequence, data owners have to trust the private key issuer when creating the corresponding private keys. In CP-ABE, instead, the approach is the contrary: the users’ private keys are associated with an attribute-set and the public key is associated with the access policy. Therefore, the confidential data owners are able to define the access policy that will entitle access to the encrypted data. CP-ABE schemes rely on Attribute Authorities (AAs) to provide each user with a private key embedding the set of attributes corresponding to that specific user. Therefore, a cornerstone of CP-ABE cryptography is a centralized TTP known as the AA. Users authenticate to the AA and request a private key associated with their set of attributes.

As dependence on a single centralized entity might be a weakness of the system and might make scalability difficult, many approaches use multiple AA entities and as a result, single-authority and multi-authority ABE schemes [51,52] can be distinguished. In a single-authority ABE scheme, there is a single central AA entity in order to manage attributes and issue the corresponding private keys to the users. In multi-authority ABE schemes, these tasks are distributed among a set of AA entities.

One of the main disadvantages of ABE cryptography, and especially of CP-ABE, is that it is highly resource consuming, and therefore, slow. This is especially critical in IoT environments, due to the resource constraints of the involved devices. Therefore, some authors [53] have proposed to use ABE to negotiate a group key in a dynamic and authenticated way. This group key will then be used to efficiently protect further communications within the group. The most expensive operation is creating a “policy tree”, and therefore, CP-ABE is especially slow in decryption operations. As a general estimation, ABE constructions are about 20 times slower than traditional asymmetric encryption. The reason for this is the execution of several pairing functions related to the specific mathematical construct used in ABE: the Weil Pairing. Additionally, the decryption operation implies the execution of a number of pairing operations that increase with the number of attributes involved in the access policy. That is why decryption is the most consuming and the slower part of ABE.

In order to reduce the decryption cost of ABE, several authors propose to implement constant attributes, therefore relying on a constant number of bilinear operations. For example, ref. [54] proposes a cost effective CP-ABE encryption scheme suitable for mobile IoT devices. In the proposed schema, both the size of secret keys and the size of ciphertexts remain constant and it does not use bilinear maps, but conventional public-key cryptosystems, which are radically cheaper from the resource consumption point of view. Similarly, in [55], the authors propose a new constant size threshold signcryption scheme based on ABE. The signcryption mechanism combines a novel attribute-based signature mechanism with the encryption mechanism in order to reduce the computational cost compared to the option of performing both operations one after the other. The proposal is oriented to protect the confidentiality and authenticity of data shared with a dynamic group of users in cloud storage environments. One of the benefits of the proposed mechanism is that the length of the signcrypted message grows linearly with the number of attributes used for signcryption. However, the mentioned approaches are based on threshold or conjunctive access policies, and therefore, the achieved expressiveness is limited.

An alternative approach to reduce decryption work on resource-deprived devices is to delegate expensive operations of the decryption process on a semi-trusted third party. This approach was first proposed by Green et al. [56] in 2011. The main idea of their proposal is to delegate most of the ABE-related decryption work in a cloud-based external entity. For this aim, the IoT device generates a transformation key from its secret key and shares it with a semi-trusted cloud server. The cloud server partially decrypts the ABE-encrypted ciphertext by using the transformation key and provides the IoT device a shorter and partially decrypted ciphertext, which the IoT device can decrypt in just one exponentiation operation.

Green et al.’s approach is similar to the proxy re-encryption concept [57,58] where an untrusted proxy is provided with a re-encryption key and it is able to re-encrypt a ciphertext encrypted with a given key $K_{1}$ with a different key $K_{2}$ without learning anything about the content of the protected message. This approach is commonly known as outsourced decryption. In this case, it must be guaranteed that the semi-trusted third party is not able to gain any information about the encrypted message during the partial decryption process and that it is not able to modify the encrypted message and provide the user with a forged version of the original message. Some approaches have been introduced to ensure these security properties [59,60,61].

The work in [62] differs from previous delegation-based proposals because it takes into account the individual context-related parameters, including the utilization level of the IoT device, the amount of available resources, the complexity of the access policy to be used and the size of the data to be encrypted. Thanks to a machine learning technique, the proposed system is able to dynamically determine if the full ABE encryption could be performed by the IoT device or if part of these tasks should be offloaded to a more powerful entity.

### 4.2. ABE as an Alternative to PKIs in IIoT

ABE or IBE schemes can be considered as an alternative to traditional PKIs that allow the reliable utilization of public key cryptography without the use of certificates. In 2015, Reimair et al. [63] identified ABE as an alternative to facing the problems of current PKIs. However, as PKIs had already been widely deployed in industry, the authors proposed a way of integrating the benefits of ABE into current PKI systems. With this aim, they proposed to replace centralized CAs by a central Security Module (SM), which verifies users’ attributes and decrypts messages for entitled users. In this way, both endpoints are able to use public key cryptography without needing to address identity management and key authentication issues. As an alternative approach to having the SM decrypting confidential information, ABE could be used to encrypt a symmetric key used to encrypt the actual confidential data. In this case, since the SM does not receive the confidential information, has a harder time in eavesdropping on confidential data.

On the other hand, as a specific mechanism to replace PKIs with ABE cryptography, the work in [64] presents a new signing and encryption scheme for IoT, which avoids the necessity of verifying public keys and storing peers’ certificates. In the proposed schema, the public key of the device corresponds to some identity information and the corresponding private key is generated by an external trusted entity known as the Private Key Generator (PKG) and communicated to the IoT device by means of a secure channel established out-of-band. For the generation of private keys, the PKG owns a system wide master secret key. The signing and encryption is performed in two steps, both of them performed by the IoT device. The first step involves most computation expensive operations and it is performed offline, before the actual message is known. The second step gathers computations that must be carried out once the message is known. The results of the performance evaluation show that it is suitable for a Raspberry Pi B with a 900-MHz Quad-core ARM Cortex-A7 CPU and 512 MB RAM.

In order to guarantee end-to-end security of data in IoT applications, the authors in [65] propose to encrypt confidential data with a symmetric key and to encrypt this symmetric key using ABE. The encrypting entity first gathers all the attributes of communicating entities and then encrypts the data with the selected attributes, depending on which destinations should read the data. In order to be able to achieve full granularity, the device identity is always an attribute. The provided performance evaluation shows that the proposed system runs adequately in a Raspberry Pi B with a 700-MHz ARM11 CPU and 512 MB RAM.

Targeting the specific Intelligent Transport Systems (ITS) context, the authors in [66] propose a hybrid security architecture for Vehicular Ad hoc Networks (VANETs) that combines PKIs with ABE and an identity manager. PKI-based encryption is used when data is sent to a single destination and ABE is used when the message is sent to a group of users. To support the proposed system, a new security entity is introduced: the Trusted Authority. This Trusted Authority is responsible for generating the ABE public parameters and the decryption keys according to the attributes claimed by users. The obtained results show that when the number of involved vehicles is greater than two, the ABE mode achieves better performance than traditional PKI-based encryption.

With a different application scenario, the authors in [67] propose a mechanism to efficiently search within data encrypted with a public key. The foundation of the proposed system are bilinear pairings, the mathematical construction that supports ABE. Confidential data is encrypted with a public key and keywords are encrypted including policy-based attributes (ABE). For the generation of the corresponding private keys, the concept of a Key Generation Center is introduced.

Based on bilinear pairings, Chien [68] proposes a solution to mitigate the problem of the aggregated communication overhead generated by the authentication processes of massively deployed IoT devices. The solution is specifically aimed at 3G and 4G networks and the fundamental concept is that each home network organizes its managed devices in groups and assigns each group an identity. Then, the authentication process is delegated to the AP, which performs it locally. The resulting solution improves scalability of authentication and key agreement in mobile networks.

Ref. [69] presents an IBE-based security architecture for IoT networks which provides authentication, authorization and accounting, using each element’s name as the public key. In this way, a reliable link is established between public keys and identities without the need of CA-signed digital certificates. The paper proposes a complex architecture model where end IoT devices are connected to Device Hosts and these Device Hosts communicate with external entities by means of Gateways. Based on this architecture, a double access control schema is proposed, where clients are first authenticated and authorized by Gateways to access a Device Host; and then, by the Device Host to access the end IoT device.

Regarding the specific industrial context, the work in [70] proposes a secure version of MQTT based on KP/CP-ABE, where the MQTT broker is in charge of providing public/private keys to publishers and subscribers after registration. Therefore, the MQTT broker gets to know the private keys of all the entities of the network.

The discussed implementations of ABE as an alternative to PKI systems are summarized in Table 4.

### 4.3. Discussion on ABE as an Alternative to Centralized PKIs

The use of ABE removes the need of identity management for the protection of confidential information. More specifically, in CP-ABE, information is encrypted according to an attribute-based access control policy, so that only destinations that own the necessary attributes are able to decrypt the encrypted data. Therefore, it removes the need to exchange CA-based certificates. However, a mechanism is needed to check the veracity of the attributes claimed by each entity, in order to assure that decryption keys are only provided to entitled destinations. This is usually performed by means of the inclusion of some central entity for attribute verification and generation of decryption keys. Additionally, in order to check the attributes owned by each entity, most frequently authentication mechanisms are used, linking attributes to authenticated identities. This authentication could be performed by distribution of symmetric shared keys within an organization, for example a factory; or by means of digital certificates, in a broader context. In the latter case, the identity management problem is moved from the resource-deprived IoT devices to the more powerful attribute authorities.

ABE is very well suited for industrial communications since it is especially adequate for communication scenarios where data are to be read by multiple destination entities. This is the case of pubsub or brokered industrial communication models, where IoT devices publish data in queues to which multiple destinations could be subscribed. In these cases, the use of ABE avoids the need of encrypting information with the public key of each intended destination. Instead, information is encrypted just once, with the appropriate attribute-based access control policy, and all destinations that match the access control policy will be able to read it.

**Figure 5:**
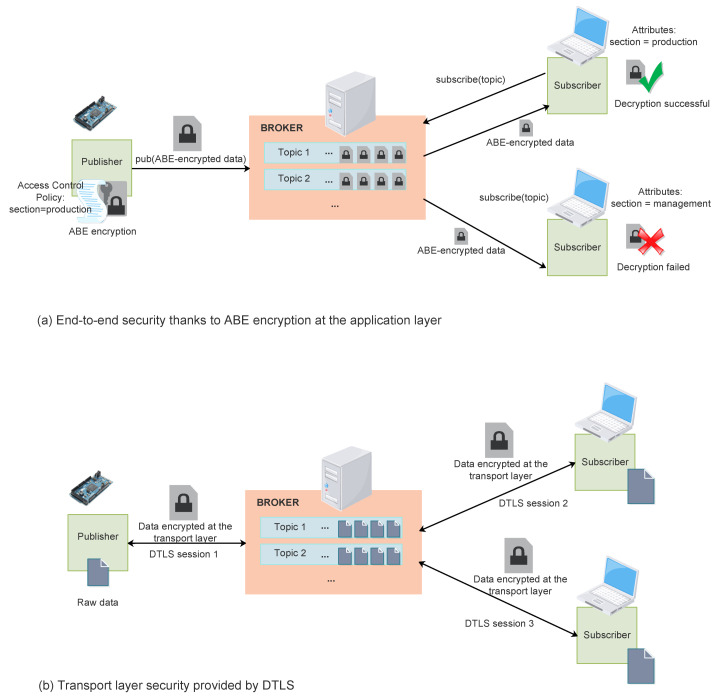
Comparison between: (**a**) end-to-end encryption provided by ABE and (**b**) transport layer encryption provided by DTLS where end-to-end confidentiality is broken at the broker.

Additionally, actual end-to-end confidentiality is guaranteed in brokered communications, as opposed to what happens when common transport layer security protocols such as TLS or DTLS are used. In fact, as TLS and DTLS sessions end at the broker, confidential information remains unprotected there, and the broker is able to access this confidential information. Conversely, since ABE is performed at the application layer, the confidential data remains encrypted even at the broker, which will not be able to decrypt it unless it complies with the access control policy. Figure 5 compares end-to-end ABE encryption to DTLS-based transport layer encryption. As shown in Figure 5a, when ABE is used, data is encrypted just once for all the intended recipients. All the recipients whose attributes match the defined encryption access control policy are able to decrypt the encrypted data. Additionally, data remains encrypted during all the stages of the transmission path, even at the broker. Instead, when transport layer security is used (as in Figure 5b), data must be encrypted for each individual transport layer connection, which results in data decryption and re-encryption at the broker. Therefore, data remains unencrypted for some time in the broker, with the security issues that it entails. Additionally, at the broker, data must be individually encrypted for each intended final recipient.

## 5. Alternatives to Current Centralized CA-Based PKIs

As already introduced, public key cryptography provides the foundations to establish reliable and scalable security mechanisms worldwide. In this regard, PKIs constitute a basic supporting pillar for the utilization of public key cryptography worldwide nowadays. However, current centralized CA-based PKIs present important problems, which are exacerbated in the case of massively deployed and resource-deprived IoT devices.

The first problem is related to the lack of trust on current centralized CAs. This lack of trust may be a consequence of security attacks perpetrated by malicious attackers or security bugs in the software run by the CAs. These problems can affect any of the different stages of certificate lifecycle management, such as certificate request, certificate signature or certificate verification. For example, in 2015, it was discovered that the ACME (Automatic Certificate Management Environment) protocol used by Let’s Encrypt to automatically issue digital certificates suffered from a security flaw which allowed potential attackers to obtain certificates for domains they did not own [71]. Moreover, in 2015, it was found out that over several years, Symantec had been issuing unauthorized test certificates for around 76 different domains without the domain owners being aware of it [72]. As a consequence, in 2018, several major platforms announced their distrust of Symantec’s public key infrastructure [73]. In 2016, several security flaws were published regarding the mechanisms used by Comodo CA to validate domains [74,75], resulting in unauthorized creation of certificates. Similarly, in 2019, Certinomis CA was found to have been issuing unauthorized certificates for different domains, leading to Mozilla removing Certinomis CA certificate from its list of trusted root CA certificates [76].

However, lack of trust of CAs goes beyond technical security issues and it is also associated with governments or corporations owning and managing CAs. Although initially the Internet was built on the concept of trust, in the post-Snowden era this is no longer the premise. In fact, CAs belong to companies or institutions, which could leverage their privileged position as trust certification authorities to attack other countries or institutions or to spy on citizens [77,78]. There have even been accusations of cyber-mercenaries acting on behalf of governments to include malicious root CA certificates in the databases of big players such as Mozilla, Microsoft, Google or Apple [79]. Furthermore, a study carried out in 2013 [80] about the HTTPS Certificate Ecosystem revealed that 99% of the CAs are concentrated in 10 countries. Additionally, regarding root CAs, just 20% was owned by commercial organizations and the other 80% belonged to religious institutions, museums, libraries and financial institutions. On the other hand, the study also found out that from a sample of 8.1 million server certificates, only 40% were valid certificates. The remaining 60% were self-signed certificates (48%), certificates signed by unknown issuers (33%) and certificates signed by untrusted CAs (19%).

Another important issue refers to scalability. According to Gartner [81], by 2025 there will be about 25,000 million IoT connections. This implies an important burden for the current centralized PKI architecture and it affects all the tasks related to certificate lifecycle management involving CAs, such as, certificate request, domain validation, certificate distribution or certificate revocation. As a result, more CAs will be created to face the ever growing number of certificates, augmenting the problem of monitoring the trust and security of CAs.

Taking into account that current centralized PKIs present important problems regarding lack of trust and scalability, alternative approaches have been proposed, which remove the necessity of centralized PKIs. On the one hand, blockchain technology has emerged as a suitable candidate to solve these issues. Its distributed nature, both regarding trust management and computation and storage, avoids the scalability and lack of trust issues inherent to centralized architectures. However, the practical implementation of blockchain technologies also implies significant performance challenges [82,83], mainly related to the storage capacity needed to keep the block chain and the computation cost of the execution of the consensus algorithm. These challenges are especially critical in the case of resource-deprived IoT devices.

### 5.1. Blockchain Fundamentals

Blockchain is a technology used to register and verify transactions in a distributed manner and following a consensus algorithm among all the participating nodes. Although blockchain is an old technology, it became very popular in 2008 when Satoshi Nakamoto started to used it as the building block of a new crypto currency: Bitcoin [84].

**Figure 6:**
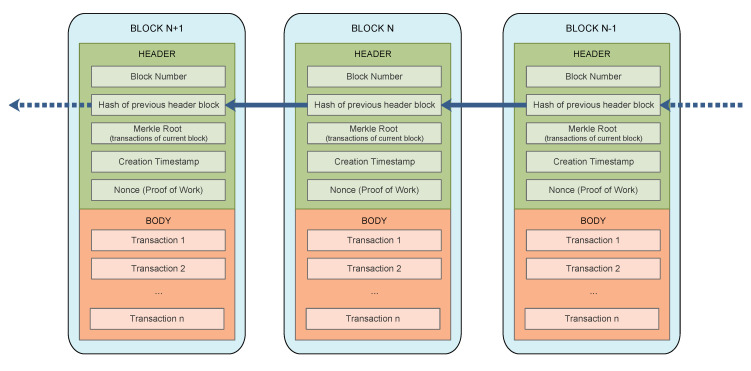
Structure of the blocks in the blockchain.

The blockchain distributed ledger consists of blocks and each block has a header and a body. The header includes information about the block, such as a timestamp and a hash of the previous header block. This hash is used to concatenate blocks in the blockchain in an immutable and unforgeable manner. If the content of a block is modified, the result of the hash computation will also change, and therefore the change will be detected. In the same way, if a block is deleted or a new block added to the chain, the hashes will also differ and the chain will be broken. On the other hand, the body of the block consists of confirmed and validated transactions. Every transaction in the block should be digitally signed with the private key of the owner, ensuring the authenticity of the transaction. The structure of the blocks that form the blockchain is represented in Figure 6.

A blockchain network might consist of two types of nodes: nodes that just read transactions and nodes that read and write transactions. The latter are known as miners. When there is a sufficiently large number of transactions (in Bitcoin 1 MB of data), they are packed together in a block. Miners verify data transactions within the block, according to defined rules, and they validate the new block by means of a consensus algorithm and the new block is stored in the blockchain. Miners are rewarded in order to validate new blocks. All of the entities participating in a transaction, as well as an important number of third parties, keep a copy of the blockchain, making it unfeasible to modify all the copies of the blockchain in order to forge a transaction.

There are many different consensus algorithms to agree on the data integrity of a block among all blockchain nodes. One of the most well-known consensus algorithms is the proof-of-work (PoW) [85], as it is the one used by the popular Bitcoin crypto currency. In this case, miners validate the legitimacy of each transaction included in a block by solving a difficult and computationally expensive mathematical problem, which cannot be solved without brute force. Therefore, miners compete to find a solution to this problem and the first one to compute a valid solution is rewarded in the blockchain. Once a valid solution is provided, its correctness can be easily verified by the rest of the participating nodes.

Another popular consensus algorithm is the Proof-of-Stake (PoS) [86]. In this case, each staker (miner) owns a wallet and some currency used in the blockchain. PoS is not as computationally expensive as PoW because miners do not compete among them to be the first to solve a complex mathematical puzzle. Instead, the network selects the nodes to validate a given block according to how many coins they own. Validating nodes are not rewarded per block, instead they take transaction fees.

Blockchains can be public or private. Public blockchains are distributed ledgers where the integrating nodes do not trust each other, while private blockchains are usually implemented within an organization and all the nodes of the blockchain are members of the organization. Among public blockchains, Bitcoin is without doubt the most renowned one. Another popular blockchain is Ethereum [87], a platform used to create any type of decentralized applications, not only related to monetary transactions. Ethereum uses Ether as its currency and makes use of smart contracts, immutable programs that allow interacting with the blockchain. Changing the state of a smart contract is priced in Ether transaction fees.

Since the blockchain grows with every new node added to it, the storage of the full blockchain implies a high storage capacity, which is not always available in the participating nodes, especially in the case of IoT devices. For example, the full Ethereum blockchain takes up to 250–280 GB in 2020. For this reason, the Light Ethereum Subprotocol (LES) was created (the size of the light node is about 100 MB) [88]. The basic idea behind LES is that it is not necessary to store the whole blockchain from the beginning block (Genesis) till the latest one in all and every node of the network. Instead, only the block header that contains an important information (the Merkle Tree) [89] is stored. This information allows to check the authenticity of the information within the blockchain.

A different approach to public distributed ledgers is IOTA [90]. IOTA differs from classical blockchains in that it does not use blocks or miners, and therefore, it does not involve high computational transactions. In fact, IOTA is based on a new approach called Tangle [91], which relies on the Direct Acyclic Graph concept. In IOTA, there is no chain and each node is a single transaction, where each edge represents a confirmation of a single transaction. In IOTA, when a new transaction arrives, it must approve two previous transactions. Therefore, users must work to approve other transactions and in this way, contribute to the network security.

### 5.2. Blockchain-Based Decentralized PKI Alternatives

In recent years, blockchains, and distributed ledger technologies (DLTs) in general, have been postulated as attractive alternatives to the centralized trust approach of current CA-based PKIs [92,93,94,95,96,97,98,99,100,101]. In fact, DLTs support a distributed trust approach which avoids the dependency on a centralized entity and improves scalability.

In [92], the author identifies the main problems of current centralized CA-based PKI architectures and analyzes the possibility of replacing them with distributed ledger technologies. After assessing different available decentralized approaches based on shared ledgers, the author concludes that the most promising solution is Ethereum [102] with proof of stake and smart contracts implemented by Smart Contract PKI (SCPKI) [103].

One of the first attempts to maintain a public ledger of users’ identities and their associated public keys is CertCoin [93]. CertCoin provides six main functionalities: registering an identity with its corresponding public key, updating the public key, searching for a public key, revoking a public key, recovering the public key corresponding to an identity and mining. Additionally, CertCoin implements some mechanisms to make the solution also available for resource-deprived devices, such as the use of cryptographic accumulators and distributed hash tables.

Similarly, the authors in [94] propose Cecoin, a distributed certificate management system based on the concepts of the Bitcoin blockchain. In Cecoin, certificates are treated as currency and stored in the blockchain, which involves three types of nodes: miners, certificate owners and certificate users. The proposed system implements mechanisms to allow certificate owners registering certificates, miners verifying the ownership of the certificates and users retrieving and validating certificates.

In a more recent work, the authors in [95] propose a new consensus algorithm in order to implement a blockchain-based distributed PKI that provides all the functions of current PKIs. The proposed algorithm allows reaching a consensus even if not all the members involved in the PKI participate in the transaction. The proposed solution has been proved to detect attacks to the system and to distinguish attacks from errors.

The work in [96] presents IoT-PKI, a decentralized PKI infrastructure for IoT based on blockchain. In the proposed system, the identity of an IoT device is bound to its public key by means of a proof of work, and a new type of distributed nodes are introduced to replace CAs and perform costly tasks on behalf of the IoT devices, such as creating a new transaction in the chain or verifying a transaction.

The authors in [97] study the suitability of blockchain-based approaches to replace current CA-based PKIs in IoT environments. Specifically, the paper considers three blockchain-based alternatives: (1) Emercoin, based on the storage of Name-Value pairs, where the ID of the certificate is stored as the “Name” field and its cryptographic hash as the “Value” pair. (2) Ethereum Smart Contracts, which allow storing more complex data structures. (3) Ethereum Light Sync Node, where the IoT node does not store the whole blockchain, but a lightweight version of the blockchain. The first two alternatives require a remote trusted blockchain node to store a copy of the blockchain on behalf of the IoT device.

Similarly, in [98], the authors propose a distributed PKI, which is based on Ethereum blockchain, as a decentralized storage for public keys. Smart contracts are used to securely update the blockchain and retrieve information from it. The proposed architecture is based on three main building blocks: (1) A Public Key Manager, which is a Network Function on the server side that authenticates the requesting user and approves the blockchain storage requests. (2) A client module, which creates an Ethereum address and securely stores a generated public key in the blockchain. (3) Smart contracts in the Ethereum blockchain implementing the necessary functions to store and retrieve the keys. For resource-constrained devices, a lightweight version is envisioned, based on LES.

The same idea has also been presented in [99], which builds on three main elements: (1) The Smart Contract, which provides functions to add new certificates to the blockchain or to retrieve the public key of a device. (2) The Device Module, which accesses the blockchain by means of an Ethereum address. (3) The Wallet Module, a Network Function executed in the server side, which authenticates the devices and approves the blockchain storage requests. The proposal assumes that each device owns a public/private key pair and the Ethereum Wallet. When an IoT device wants to add a new public certificate to the BlockChain, it calls the *addDevice* function in the SmartContract by signing a transaction. Then, the device module sends an approval request to the Wallet Module and the device is approved by the manager of the system. Hereafter, every other device can call the *getDevice* function to obtain the public key of the device.

The work in [100] presents an alternative approach based on CertCoin to support very resource-deprived devices, while preserving their privacy, where full nodes are introduced to aid in specific functions. These full nodes are considered honest but curious. Therefore, in order to preserve the privacy of the thin clients’ searches, a Private Information Retrieval-based search mechanism is proposed. In short, the ID of the entity whose public key is wanted is mixed up with other IDs forming a *d*-dimensional cube, and being the position of the desired ID randomly selected. In this way, at least *m* full nodes need to collude to be able to compute the position of the desired ID.

Finally, in a work specifically focused on vehicular networks [101], a PKI based on the IOTA distributed ledger is proposed. The authors propose to enhance the SECMACE VPKI [104], a PKI for vehicular networks, which matches the security architecture of Cooperative Intelligent Transport Systems (C-ITS) agreed by most standardization bodies, by means of the integration of a distributed ledger. More specifically, each CA in SECMACE is provided with a IOTA wallet and the corresponding seed, which also allows to use the Masked Authentication Message (MAM) channels used to implement secure communications for certification registration and update procedures.

Table 5 summarizes the studied alternatives to implement a distributed PKI system based on distributed ledgers.

### 5.3. Discussion on Blockchain-Based Distributed Alternatives to Centralized PKIs

Blockchain-based PKIs have been proposed as an alternative to CA-based PKIs in order to tackle the issues associated with the centralized nature of traditional PKIs, namely, lack of trust of the centralized CAs and limited scalability. In blockchain, networks’ trust is acknowledged in a distributed manner, following a consensus algorithm. Additionally, as the blockchain is replicated in a number of independent nodes, it is not possible for a malicious entity to change the content of a blockchain node or add or remove blocks.

However, blockchain-based approaches still present important challenges when they are to be implemented in IoT networks, mainly due to the big size of the blockchain and the high computational cost of the consensus algorithm. With respect to the high storage capacity required in the participating nodes, which is not available in IoT devices, light versions of different blockchains are emerging, which basically consist of not storing the whole blockchain in each IoT device. Regarding the computation capacity needed to execute the consensus algorithms, it exceeds the capacity available in common IoT nodes, and the most common solution is to introduce resource-rich nodes in order to support IoT devices in these tasks, that is, to act as miners. However, this again raises issues about the shared trust with the resource-rich node and the necessity of implementing secure communication mechanisms. Additionally, the common Proof of Work consensus algorithm is not deemed suitable for IoT networks, and alternative consensus algorithms such as proof of stake are considered more suitable.

In the context of an industrial IoT scenario, it is easy to find resource-rich nodes which could aid IIoT devices in the storage of the full blockchain and the execution of the consensus algorithm. These tasks could be delegated to a smart-node in the edge of the IIoT network, such as the IIoT border gateway, or a specific node could be included for this purpose. In any case, independent security mechanisms should be implemented to protect the communications between each IIoT device and the resource-rich device aiding in blockchain functions. In this regard, the most common practice is to deploy symmetric shared keys within the industrial plant in order to protect internal communications between the different entities belonging to the organization.

On the other hand, smart contracts have been identified as a useful mechanism to interact with blockchains, since they allow to store in the blockchain data structures more complex than simple name-value pairs. Additionally, these high level programs allow to implement mechanisms such as access control to query or update the blockchain.

## 6. Comparative Analysis of Different Proposed Approaches

**Figure 7:**
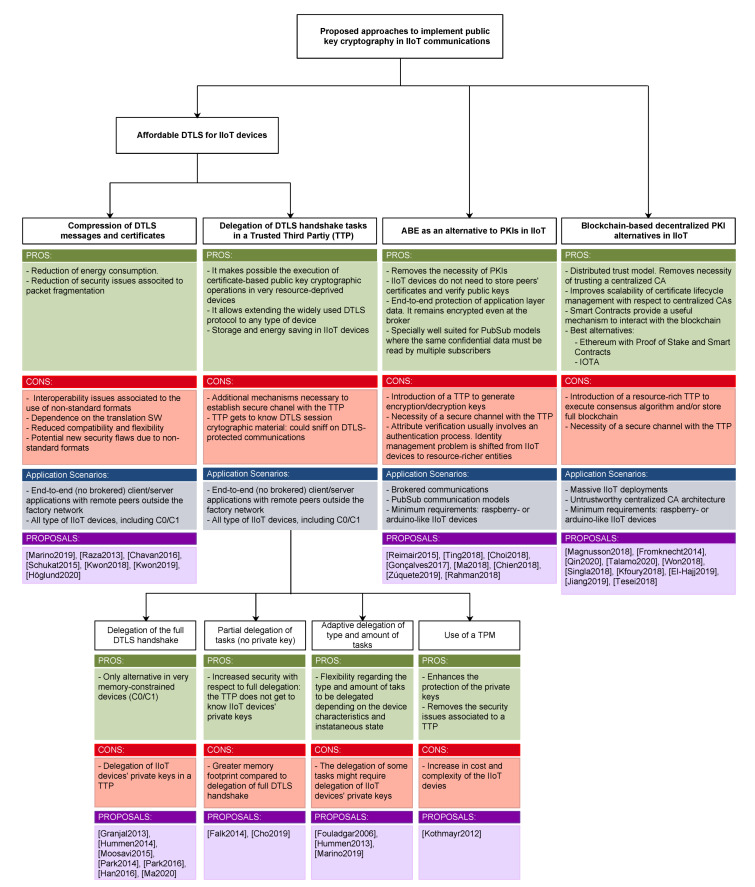
Comparison of pros & cons and different application scenarios of the analyzed approaches [21,22,23,24,26,27,28,30,31,32,33,34,35,36,39,40,41,42,44,63,64,65,66,67,68,69,70,92,93,94,95,96,97,98,99,100,101].

This paper presents the current challenges to implementing certificate-based public key cryptography in IIoT scenarios and surveys the different alternatives that have been proposed so far to address these issues. Table 6 provides a matching between the identified challenges and the different approaches proposed to tackle them. Figure 7, in turn, provides an in-depth comparison of the different proposed approaches detailing their pros and cons, and specifying the application scenarios where they fit.

Most industrial communication protocols build their secure version over a TLS or DTLS layer. Although both TLS/DTLS support alternative authentication mechanisms, such as Pre-Shared Master Keys (PSMKs) and Raw Public Keys (RPKs), certificate-based authentication is recognized as the most robust and scalable solution [105]. This results in the necessity to accommodate current highly resource consuming certificate-based operations and PKIs to the scarce resources of the IIoT devices involved in the communications. The proposed approaches are based on delegating most resource consuming tasks to resource-richer devices and to compress DTLS messages and X.509 certificates. Compression reduces the amount of bytes to be transmitted and received by IIoT devices, reducing in turn energy consumption and fragmentation.

The delegation of DTLS handshake tasks is sometimes the only feasible approach to enable the participation of IIoT devices in end-to-end client/server communications with entities outside the factory domain. It only makes sense to delegate DTLS tasks when the communication is end-to-end between an IIoT device and an external entity. In other cases, such as a brokered communication, it would be preferable to opt for a PSK-based DTLS session between the IIoT device and the broker, and a certificate-based DTLS session between the broker and the external entity.

Full DTLS handshake delegation achieves very little memory footprints, but it implies the delegation of the IoT device’s private key. Therefore, it is justified in very constrained IoT devices, such as C0-C1 motes, which are not able to perform the handshake by themselves. If the involved IIoT devices are more powerful (raspberry- or arduino-like), then a partial delegation of DTLS tasks is a preferable option. This solution allows to save storage, energy and processing resources in the IIoT device by outsourcing some heavy tasks, but without the security concerns associated with making known to a third party the private keys of IIoT devices. The most suitable approaches are those that allow to flexibly select how many and which tasks to delegate depending on the type of device and its instantaneous state. In such cases, the network administrator could decide on the trade-off between security and performance for each specific use case. In any case, as previously explained, if the TTP negotiates the cryptographic material to be used in the DTLS session, it has all the required knowledge to sniff in the subsequent DTLS communications protected with that cryptographic material.

A more straightforward type of delegation and one which enhances the security level of the solution is to integrate in the IIoT devices a specific security module, such as a TPM. This module stores in a more robust way all the cryptographic keys and performs cryptographic computations more efficiently. However, in most industrial scenarios, the integration of a TPM in each IIoT device is out of the scope, due to the increase in complexity and cost.

Additionally, compression mechanisms are presented as a way of saving IIoT devices’ resources, especially storage and battery, in DTLS communications. These mechanisms are complementary to the delegation mechanisms and allow to reduce the size of the DTLS protocol headers and of the X.509 certificates, mainly by using non-standard formats. The reduction of the size of messages results in less fragmentation, and therefore, removes the security vulnerabilities associated with packet fragmentation. The more natural place to perform the translation between compressed and standard messages is the IIoT gateway at the boundary of the IIoT network. However, the use of non-standard protocols and messages is not recommended in industry since it hinders interoperability and flexibility. Additionally, non-standard protocol messages could be subject to new unknown security flaws.

On the other hand, ABE stands out as a suitable mechanism to guarantee end-to-end confidentiality in brokered PubSub communication models, which are very frequent in industrial scenarios. In such cases, DTLS encryption at the transport layer is not enough to protect the confidentiality of the transmitted data end-to-end, since the transport layer session ends at the broker, and application layer encryption, such as ABE, is necessary. As ABE integrates an attribute-based access control policy in the encryption process, it allows the publisher to encrypt the information just once and any subscriber owning a decryption key that matches the encryption policy will be able to decrypt it. Additionally, as ABE encryption is based on attributes, it removes the need of IIoT devices to manage identities and therefore, to deal with certificates.

However, ABE encryption is resource-expensive and cannot be performed by the most severely constrained IIoT devices, such as C0-C1 motes. At least arduino- or raspberry-like capacities are required. Additionally, the use of ABE involves new challenges related to the secure and trustworthy management of attributes. For this aim, a TTP is usually introduced to authenticate attributes and generate decryption keys. The authentication of attributes is frequently based on identity authentication and therefore, the identity-management problem is shifted from resource-deprived IIoT devices to resource-richer TTPs.

Finally, current PKIs also suffer from lack of trust on the centralized CAs and difficult scalability. Thanks to its distributed nature, the blockchain technology presents a suitable alternative to address these issues. However, participating in a blockchain implies high resource consumption regarding storage capacity, in order to store the blockchain, and processing, to execute the consensus algorithm. IIoT devices are not able to meet these needs, not even the most powerful ones, such as Raspberry Pi 2 Model B with a 900-MHz Quad-core ARM Cortex-A7 CPU and 1 GB RAM. For this reason, the implementation of distributed ledgers in IIoT networks, necessarily implies the delegation of most consuming tasks, such as blockchain storage and mining, to resource-richer devices, while the IIoT devices are integrated as light nodes. In industrial scenarios, some other entities of the industrial plant could support the IIoT devices by implementing the full blockchain nodes that aid the light IIoT nodes in the validation of the transactions and the storage of blocks. All in all, the Ethereum blockchain with the proof of stake consensus algorithm and smart contracts’ support has been recognized as the most suitable approach to implement decentralized PKIs in IoT networks. The IOTA network has also been identified as a promising approach, but it is a novel development with not as much support available yet.

It must be noted that all the proposed approaches rely on a centralized third party to aid the IIoT devices in the corresponding operations: in the case of the DTLS delegation- and compression-based approaches, this third party is the entity where certain tasks are delegated or where the translation between compressed and standard formats is performed. In the case of ABE-based approaches to replace traditional KPIs, the third party is responsible for attributes’ validation and for the generation of decryption keys. Finally, in blockchain-based approaches, the third party aids IIoT devices in tasks that exceed their capacity such as storage of the full blockchain or execution of the consensus algorithm. Therefore, new security concerns arise regarding the protection of the communications between the IIoT devices and the third party, especially when it must be a trusted entity. This issue is usually solved by the out-of-band distribution of PSKs within the factory domain.

## 7. Conclusions

This paper surveys the alternatives proposed in the literature so far to tackle the challenges raised by the use of certificate-based public key cryptography in IIoT scenarios. The identified challenges cover a wide scope of issues related to resource limitations of IIoT devices, scalability, long lifetime of industrial systems, one-to-many communication models and lack of trust in centralized CAs. Therefore, different types of solutions have been proposed in the literature, each addressing specific challenges. The proposed solutions are complementary and they depend on the application scenario and the characteristics of the involved IIoT devices.

The main goal of this paper is to provide a matching between the identified challenges and the surveyed solutions, as well as an in-depth classification and analysis of the existing alternative approaches, detailing their pros and cons, and specifying the application scenarios where they fit. The final aim is to provide IIoT network designers and administrators with a useful tool to select the best approach to secure their specific deployment.

## Figures and Tables

**Table 1:** Summary of industrial communication protocols.

	AMQP 1.0	MQTT	XMPP	OPC UA	Modbus TCP	CoAP
**Year**	2011	1999	1999	2006	1979	2010
**Communication Architecture**	Peer-to-peer or brokered	Brokered: client devices publish/subscribe in the server (broker)	Brokered: client devices publish/subscribe in the server (broker)	Client-server: clients (HMI, SCADA) directly query servers (industrial devices)	Master/slave: Masters (HMI, SCADA) directly query slaves (industrial systems)	Client-server (IoT devices might act as clients or servers). Multicast communications also supported
**Communication Model**	PubSub (push or pull)	PubSub (push)	PubSub (push)	Two alternatives: - PubSub (push). - Query/response	Request/response	Request/response
**Intermediary Dntity**	Optional broker (queues and bindings to distribute messages to queues). The broker is mandatory in previous versions	Broker (Server)	XMPP server.XMPP GW to translate to other messaging protocols	✗	✗	Optional: proxies, caching, gateways to other protocols, etc.
**Transport Layer**	TCP, UDP and SCTP	TCP, UDP and SCTP	TCP	SOAP, HTTP, HTTPS, TCP, UDP, etc.	TCP	UDP
**Security**	TLS/DTLS and X.509 certificates for peer authentication: encryption only or encryption and authentication.Extensible authentication: SASL (anonymous, plaintext, diggest-MD5, etc.)	TLS/DTLS and X.509 certificates for client/server authentication	TLS and X.509 certificates for client-server authentication.Extensible authentication: SASL (plaintext, diggest-MD5, Kerberos etc.)	Transport Layer: - Based on PKIs and X.509 certificates. - Sign or sign and encrypt. Application layer: - over the secure channel established in the trasport layer. - End user authentication based on tokens (depending on authentication mechanism used) - Group keys (one publisher, many subscribers)	TLS and X.509 certificates. Peer authentication and authorization, by means of roles conveyed in certificate extensions.Frame confidentiality and integrity	DTLS and X.509 certificates. Peer authentication and frame integrity and confidentiality. OSCORE for end to end confidentiality and authenticity

**Table 2:** Summary of DTLS handshake delegation based approaches.

Author	Delegated Operations	Delegated on	Security with Delegator	Private Key Owned by Delegator	Session Key Known by Delegator	Memory Footprint	Targeted Resource
Granjal et al. [21]	Full DTLS handshake	6 LoWPAN Border Router	PSKs supported by Access Control Server	✓	✓	RAM: 0.3 KB ROM: 9.7 KB	C0–C1
Hummen et al. [22]	Full DTLS handshake	Delegation server	PSKs	✓	✓	RAM: 3.42 KB ROM: 14.17 KB	C0–C1
Moosavi et al. [23]	Full DTLS handshake	IoT network gateway	PSKs	✓	✓	RAM: 3.51 KB ROM: 14.29 KB	C1–C2
Park et al. [24]	Full DTLS handshake	Secure Service Manager (powerful server)	PSKs	✓	✓	Not Specified	C0–C1
Park et al. [26]	Full DTLS handshake	Virtual thing in cloud	PSKs	✓	✓	RAM < 10 KB ROM: 10.16 KB	C0
Han et al. [27]	Full DTLS handshake and record	Back-end offload (powerful device in the IoT network)	PSKs	✓	✓	Not Specified	C0–C1
Ma et al. [28]	Diffie–Hellman key exchange and certificate validation	SDN controller	Out-of-band mechanisms, e.g., PSKs	✓	✓	Not Specified	C1
Falk et al. [30]	Certificate validation	centralized server	Not specified	✗	✗	Not Specified	Not Specified
Cho et al. [31]	Partial DTLS handshake	Security agent (powerful node)	PSKs	✗	✓	RAM: 14–47 B ROM: 73–99 B (depends on implemented HW)	C2–C2++
Fouladgar et al. [32]	Version 1: authentication Version 2: whole TLS handshake	IoT network gateway	PSKs	Version 1: no Version 2: yes	Version 1: no Version 2: yes	Not Specified	C0–C1
Hummen et al. [33]	Version 1: validation of certificate chain Version 2: full DTLS handshake	Version 1: IoT network gateway Version 2: resource-rich TTP	PSKs	Version 1: no Version 2: yes	Version 1: no Version 2: yes	Not Specified	Version 1: raspberry-like Version 2: C0–C1
Marino et al. [34]	Flexibility to delegate: - ECDH keys generation - Premaster secret generation - ECDSA signature generation - ECDSA signature verification - Certificate verification	PKIoT server (powerful node)	Out-of-band mechanisms, e.g., PSKs	Depends on the delegated operations	Depends on the delegated operations	Not Specified	C2++
Kothmayr et al. [35]	Certificate storage and all public key cryptography operations	Trusted Platform Module (TPM)	Hardware (chip integrated in IoT device)	✓	✓	Not Specified	C0–C1

**Table 3:** Summary of compression-based approaches.

Author	Compressed Elements	Compression Mechanisms	Compatibility with Current PKIs	Achieved	Targeted Resource
Raza et al. [36]	DTLS handshake and record protocol headers	New bit sequence for the 6LoWPAN NHC	✓	62–75%	C2–C2++
Chavan et al. [39]	DTLS *ClientHello*, *ServerHello* and *Record* protocol headers	6LoWPAN NHC	✓	58–75%	C2–C2++
Schukat et al. [40]	X.509 certificates	Self-descriptive card verifiable certificates and avoid ASN.1 encoding	✗	Not Specified	Not specified
Kwon et al. [41,42]	X.509 certificates	CBOR encoding and removal of fields with implicitly known values	Compression/reconstruction at the IoT border router	37%	C2–C2++
Marino et al. [34]	X.509 certificates	Replaced by URI to the full certificate	✗	70%	C2
Hoglund et al. [44]	X.509 certificates	CBOR encoding and removal of fields with constant values according to the defined profile	Compression/decompression at the 6LoWPAN border router	>50%	C2–C2++

**Table 4:** Summary of approaches based on using ABE as an alternative to PKIs.

Author	Encryption Mechanism	Encryption Content	Decryption	Security with Intermediary Third Party	Targeted Resource
Reimair et al. [63]	IBE/ABE	Confidential message or symmetric key used to encrypt confidential message	Performed by the security module	Not specified	PCs or Smart phones (HW specifications not detailed)
Ting et al. [64]	IBE	Confidential message	Generated by a TTP: Private Key Generator	Secure channel established by offline methods	Raspberry Pi B with a 900-MHz Quad-core ARM Cortex-A7 CPU and 512 MB RAM
Choi et al. [65]	CP-ABE	Symmetric key used to encrypt confidential message	Attribute certificates issued by an IoT CA	Secure channel established by offline methods	Raspberry Pi B with a 700-MHz ARM11 CPU and 512 MB RAM
Gonçalves et al. [66]	ABE	Symmetric key used to encrypt confidential message	Decryption keys generated by the Trusted Authority	Communications with the Trusted Authority (TA) secured by means of certificate-based public key communications. Nodes are pre-loaded with the TA’s certificate.	Personal Computer with an Intel Core i7 and 16 GB RAM
Ma et al. [67]	CP-ABE	Keywords describing data stored in cloud	Partial private keys generated by a TTP: Key Generation Center	No secure channel needed	Dell PC with an I5-4460S 2.90-GHz processor, 4 GB RAM and Windows 8 operating system
Chien [68]	Bilinear pairing-based cryptography	Authentication keys	Private key generated and owned by the Registered Home	Not needed	3G/4G-enabled devices. HW characteristics not specified
Zquete et al. [69]	IBE	Authorization ticket issuing protocol messages	Private keys generated and owned by each device	Not needed	Not Specified
Rahman et al. [70]	ABE	MQTT messages	Private keys generated by MQTT broker	Not specified	Arduino Uno microcontroller board based on ATmega328P

**Table 5:** Summary of approaches based on distributed ledgers.

Author	General Architecture	Content of the Transactions	Use of Smart Contracts	Allowed Operations	Targeted Resource
Magnusson [92]	Ethereum blockchain in IoT devices	Not specified	✓SCPKI	Not specified	Raspberry Pi 2 Model B with a 900-MHz Quad-core ARM Cortex-A7 CPU and 1 GB RAM
Fromknecht et al. [93]	Public blockchain to register domains and their corresponding public keys	Users’ identities and public keys	✗	Register, update, search and revoke identity/public key pairs	Not specified
Qin et al. [94]	Blockchain with three types of nodes: miners, certificate owners and certificate users	Address, domain and certificate	✗	Register, revoke, renew and identity assignment	Not specified
Talamo et al. [95]	Novel consensus algorithm implemented in a blockchain for certificate validation	Certificate verification query	✓	Certificate validation	results based on simulation where heterogeneous CPUs and PC components are considered
Won et al. [96]	Distributed B-nodes replace centralized CAs and create/verify transactions on behalf of IoT devices. The identity of an IoT device is bound to its public key by means of a proof of work. Each IoT device is preconfigured with at least one trusted B-node.	Type of operation, identity and signature	✗	Register, update, revoke	Raspberry Pi 2 Model B with a 900-MHz Quad-core ARM Cortex-A7 CPU and 1 GB RAM
Singla et al. [97]	Three alternatives to replace centralized CAs with blockchain: (1) Emercoin, (2) Ethereum Smart Contracts and (3) Ethereum Light Sync Nodes	Option 1: certificate IDs and their cryptographic hashes as Name-Value pairs. Option 2 and 3: device ID, device owner, cryptographic hash of the certificate and validity of the certificate	In option 2: Ethereum Smart Contracts	Option 1: registration. Option 2 and 3: add a device, remove a device, get the hash of a device	Raspberry Pi 2 with a 900-MHz 32-bit ARM Cortex-A7 CPU and 1 GB RAM
Kfoury et al. [98]	Ethereum blockchain as decentralized storage of public keys, and smart contracts for secure interaction with the blockchain	Client ID, Client public key, token identifying the client module	✓	Add client, get client, approve client	Not specified
El-Hajj et al. [99]	Ethereum blockchain as decentralized storage of public keys, and smart contracts for secure interaction with the blockchain	Not specified	✓	Add client, get client, approve-client	Not implemented. Arduino and raspberry-like devices envisioned
Jiang et al. [100]	CertCoin with privacy-preserving search mechanism for thin clients that rely on full nodes	Users’ identities and public keys	✗	Privacy-preserving search for identities/public keys	Not specified
Tesei et al. [101]	IOTA for the distributed storage of public keys integrated in SECMACE VPKI	Identities and certificates	✗	Not specified	Not specified

**Table 6:** Issues of public key cryptography in IIoT scenarios addressed by the different proposed approaches.

Challenges to Apply Public Key Cryptography in IIoT Scenarios	Delegation of DTLS Handshake Tasks to a TTP	Compression of DTLS Messages and Certificates	ABE as an Alternative to PKIs	Blockchain-Based Decentralized PKIs
Resource limitations of IIoT devices	✓	✓		
Long lifetime of industrial systems (obsolete SW)	✓			
Necessity to encrypt for multiple destinations in PubSub communication models			✓	
In brokered communications, transport layer security ends at the broker			✓	
Lack of trust in centralized CAs				✓
Massive deployment of IIoT devices				✓
